# The immunomodulatory effect of lithium as a mechanism of action in bipolar disorder

**DOI:** 10.3389/fnins.2023.1213766

**Published:** 2023-08-17

**Authors:** Łukasz P. Szałach, Katarzyna A. Lisowska, Wiesław J. Cubała, Margherita Barbuti, Giulio Perugi

**Affiliations:** ^1^Department of Pathophysiology, Faculty of Medicine, Medical University of Gdańsk, Gdańsk, Poland; ^2^Department of Psychiatry, Faculty of Medicine, Medical University of Gdańsk, Gdańsk, Poland; ^3^Psychiatry Unit 2, Department of Clinical and Experimental Medicine, University of Pisa, Pisa, Italy

**Keywords:** bipolar disorder, lithium, cytokines, apoptosis, lymphocytes, immune system

## Abstract

Bipolar disorder (BD) is a chronic mental disorder characterized by recurrent episodes of mania and depression alternating with periods of euthymia. Although environmental and genetic factors have been described, their pathogenesis is not fully understood. Much evidence suggests a role for inflammatory mediators and immune dysregulation in the development of BD. The first-line treatment in BD are mood-stabilizing agents, one of which is lithium (Li) salts. The Li mechanism of action is not fully understood, but it has been proposed that its robust immunomodulatory properties might be one of the mechanisms responsible for its effectiveness. In this article, the authors present the current knowledge about immune system changes accompanying BD, as well as the immunomodulatory effect of lithium. The results of studies describing connections between immune system changes and lithium effectiveness are often incoherent. Further research is needed to understand the connection between immune system modulation and the therapeutic action of lithium in BD.

## 1. Introduction

Bipolar disorder (BD) is a severe mental disorder traditionally characterized by recurrent episodes of mania and depression alternating with periods of euthymia. Around 1% of the global population is estimated to suffer from the disease regardless of nationality or economic status (Grande et al., 2016). BD is a complex, multifaceted, and heterogeneous condition with frequent comorbid symptoms or diagnoses (Grande et al., 2016). In addition, many BD patients experience symptoms of opposite polarity during a mood episode and symptoms of mania (BP-I)/hypomania (BP-II) and depression during periods of euthymia (Stahl et al., 2017). Finally, symptoms of manic or depressive episodes cluster differently in different subjects and even in the same subject across different episodes. The course of the disease, frequency, length, and severity of episodes varies significantly and, if not treated, significantly impairs the patient’s life. Bipolar disorders substantially reduce psychosocial functioning and are associated with losing approximately 10–20 potential years of life (McIntyre et al., 2020). More than 70% of patients relapse after the first episode. The number of relapses ranges from 2 to 30. Moreover, up to 20% of BD patients commit suicide (McIntyre et al., 2020).

Long-term treatment’s primary goal is to reduce mood episodes’ frequency, shorten the duration of episodes, prolong euthymic periods, and improve patients’ overall quality of life by reducing most, if not all, disease symptoms. The first-line treatments are mood-stabilizing agents, such as lithium (Li) and anticonvulsants (valproate, carbamazepine, and lamotrigine). However, the mechanism of pharmacological treatment for BD is not completely clear. In particular, despite being a simple chemical element, Li is highly effective in managing mania, depression, and the prophylactic treatment of BD patients, preventing both manic and depressive episodes (Fountoulakis et al., 2022).

The vast heterogeneity of BD clinical presentations complicates the research on the underlying pathogenesis and the definition of standardized treatment approaches. The neurobiology of BD is not fully understood, although environmental and genetic factors are known to play a role in its pathogenesis (Sigitova et al., 2017). Much evidence suggests that inflammatory mediators and immune dysregulations may play an important role in the pathogenesis of BD. Moreover, it seems that the role of Li in BD therapy is not limited only to the modulation of neurological pathways but may also affect inflammatory and immunological processes that play a significant role in the development of the disease.

In this review, we discussed the role of inflammatory and immune mechanisms in the development of BD. In addition, we present the most recent human studies describing the immunomodulatory properties of Li that may be relevant to the course of BD.

## 2. Methods

**Figure 1:**
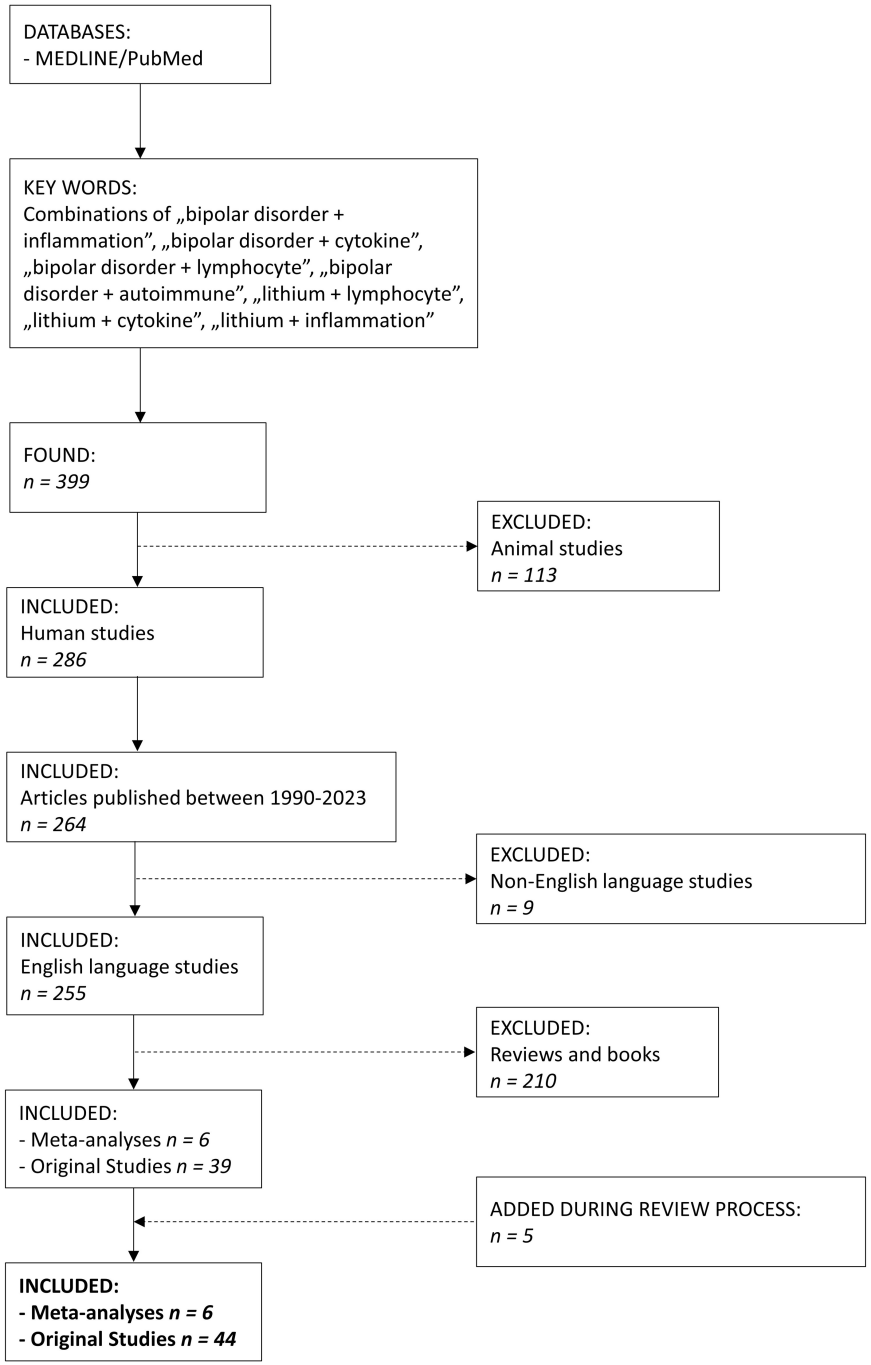
The flowchart of the article selection process.

We searched MEDLINE/PubMed database using a combination of keywords “bipolar disorder + inflammation,” “bipolar disorder + cytokine,” “bipolar disorder + lymphocyte,” “bipolar disorder + autoimmune,” “lithium + lymphocyte,” “lithium + cytokine,” “lithium + inflammation” (Figure 1). We found 399 articles. The search was narrowed down to the years 1990–2023. After excluding animal studies, non-English studies, reviews and books, we included 50 papers, 6 meta-analyses and 44 original studies. The article presents the latest research documenting the immunomodulatory properties of Li in humans.

## 3. Mechanism of action of lithium

Lithium seems to affect many biochemical pathways within neurons. One of the best-known lithium mechanisms of action is the inhibition of glycogen synthase kinase-3 (GSK-3; Iwahashi et al., 2014). This enzyme is involved in a wide range of signal transduction pathways (Cui et al., 1998) and influences the metabolism, proliferation, differentiation, development, and apoptosis of central nervous system (CNS) cells, where it is highly expressed (Morgan-Smith et al., 2014). Since GSK-3 has mainly a proapoptotic action, its inhibition by Li may lead to neuroprotection, long-term plasticity, and mood stabilization (Gould et al., 2004).

In addition, lithium affects the intrinsic apoptotic pathway regulated primarily by the Bax protein and downregulated by the anti-apoptotic protein Bcl-2. Long-term Li treatment decreases p53 and Bax protein production and significantly augments transcription of the Bcl-2 gene, resulting in a neuroprotective effect (Chen and Chuang, 1999; Manji et al., 2000). The anti-apoptotic effect of lithium can also be achieved by regulating oxidative stress due to increased anti-oxidant levels and decreased lipid peroxidation levels (de Sousa et al., 2014).

Another well-proven lithium effect is the influence on the phosphatidylinositol cycle. Li reduces the levels of inositol in CNS cells by inhibiting inositol monophosphatase. The “inositol depletion” hypothesis should explain the therapeutic effect of lithium through the reduction of metabolism in overactive neurons. In addition, other second messenger pathways, including cAMP and G-protein pathways, are influenced by Li (Agranoff and Fisher, 2001; Alda, 2015).

Lithium might also play a role in modulating neurotransmission. Chronic administration of lithium causes an increase in glutamate reuptake, decreasing its concentration in the synaptic cleft and thus preventing its excitotoxic effect (Zanetti et al., 2014). Li also reduces post-synaptic activation of NMDA receptors due to decreased phosphorylation (Hashimoto et al., 2002), reducing the overexcitation of post-synaptic neurons and resulting in an anti-manic action (Jope, 1999). In addition to glutamate, lithium reduces excessive dopaminergic activity in the prefrontal cortex in animal manic models (Ago et al., 2012). It also enhances cholinergic activity, which effects in reducing depression-like behavior in mice (van Enkhuizen et al., 2015), as well as GABAergic activity (Jope, 1999) and serotonergic activity by inhibiting the autoreceptors 5-HT1a (serotonin 1A receptor) and 5-HT1b (Massot et al., 1999).

Circadian rhythm abnormalities were associated with BD. Recently, the influence of lithium on circadian rhythm genes has been studied (Mukherjee et al., 2010). It has been shown that lithium modulates clock gene expression, such as PER or ROR (McCarthy et al., 2013). What is more interesting, lithium’s response might depend on certain clock gene variations (McCarthy et al., 2012).

## 4. Autoimmune diseases and bipolar disorder

Much evidence suggests a role for inflammatory mediators and immune dysregulations in the pathogenesis of psychiatric disorders and highlights several common features between BD and autoimmune diseases (Eaton et al., 2010; Berk et al., 2011; Rege and Hodgkinson, 2013). For example, autoimmune diseases are multifactorial disorders related to an interaction between genetics and the environment (Ellis et al., 2014) and often exhibit familial aggregation (Beaulieu, 2012). Moreover, autoimmune reactions often progress much more slowly than immune reactions to pathogens, suggesting that control mechanisms can continue to work until a threshold is crossed, after which symptomatology progresses from subclinical to clinically significant. Finally, control mechanisms may temporarily restore antigenic tolerance in autoimmune diseases, leading to a cyclic disease exacerbation and remission pattern.

Mood symptoms have increased prevalence in various inflammatory conditions, including autoimmune diseases, cardiovascular diseases, diabetes, obesity, metabolic syndrome, and more benign inflammatory conditions such as asthma and allergies (Brydon et al., 2009). In a recent Italian clinical study, a high prevalence (48.1%) of autoimmune-allergic diseases was observed in 347 BD patients, with no differences in gender distribution (Perugi et al., 2015). A large Danish cohort study showed that a history of Guillain-Barré syndrome, Crohn’s disease, and autoimmune hepatitis was associated with a raised risk of BD (Eaton et al., 2010). The authors concluded that autoimmune processes precede the onset of BD. A subsequent study based on Danish hospital data reported that many autoimmune diseases and infections requiring hospitalization increase the risk of developing schizophrenia and mood disorders (Benros et al., 2011). These epidemiological and clinical observations suggest a possible immunological contribution in some subgroups of patients with severe mental disorders, such as BD and other psychotic disorders. However, whether it is a causal relationship or an epiphenomenon due to other environmental factors or common genetic vulnerability remains to be demonstrated.

Inflammatory mechanisms can affect the CNS through many pathways (Dalman et al., 2008; Yolken and Torrey, 2008; Eaton et al., 2010). Peripheral inflammation influences the CNS without crossing the blood–brain barrier (BBB) through pro-inflammatory cytokines that activate the tryptophan-kynurenine pathway, regulate serotonin production and N-methyl-D-aspartate (NMDA) receptor activity, and may also indirectly affect dopamine regulation (Dantzer et al., 2008). In addition, increased inflammation in autoimmune diseases may cause increased permeability of BBB, making the CNS vulnerable to immune components, such as cytokines and auto-antibodies.

Although the link between stress and mood disorders is well recognized (Weiss et al., 1994), no specific studies examine the role of stress, immune dysregulation, and autoimmunity in BD. However, retrospective studies have found that up to 80% of patients with the autoimmune disease report uncommon emotional stress prior to disease onset (Stojanovich and Marisavljevich, 2008), and it has been suggested that immune activation may vary in BD patients according to different affective states (Ortiz-Domínguez et al., 2007).

## 5. Immune system alterations in bipolar disorder

### 5.1. Changes in serum cytokines

BD patients exhibit several immunological changes during depressive and manic episodes. A meta-analysis by Modabbernia et al. (2013) showed that anti-inflammatory and pro-inflammatory cytokines were elevated in the serum of BD patients compared with healthy subjects, underlining the hyperactivity of the immune system. Authors confirmed that BD patients are characterized by increased serum interleukin 4 (IL-4), IL-10, and tumor necrosis factor-alpha (TNF-α), soluble IL-2 receptor (sIL-2R), sIL-6R, and soluble TNF receptor 1 (sTNFR1). Additionally, specific changes in the cytokine levels were observed during manic and depressive episodes. IL-1 receptor antagonist (IL-1RA), sIL-2R, and sTNFR1 were significantly higher in patients with mania, while IL-10 levels were significantly higher in depressed patients (Modabbernia et al., 2013). Also, euthymic patients had high IL-1RA levels compared to healthy control.

Another summary of existing studies investigating different episodes of BD also demonstrated significant exacerbation of the immune system and confirmed some of the findings described above (Goldsmith et al., 2016). Compared to healthy people, BD patients with acute manic episodes had significantly increased serum IL-6, TNF-α, IL-1RA, and sIL-2R. Following treatment for acute mania, there was a significant decrease in IL-1RA levels. Euthymic patients were characterized by a moderate increase in IL-1β, IL-6, IL-10, and sTNFR1, and a more significant increase in IL-4, sIL-2R, and sIL-6R compared to healthy control (Goldsmith et al., 2016). However, the authors could not demonstrate characteristic changes specific to BD – similar results were obtained for acutely ill patients with schizophrenia and major depressive disorder (MDD). These results indicated that these disorders might share common underlying immune dysfunction pathways. Meanwhile, the latest meta-analysis by Zhang et al. (2023) showed that IL-10 is significantly increased in BD patients compared to MDD patients, while the level of IL-1β was significantly decreased. In their meta-analysis, Solmi et al. (2021) demonstrated that IL-6 and TNF-α but not IL-1β were elevated in BD patients compared with healthy control. Also, the serum TNF-α was elevated in patients with depressive or manic episodes but not in euthymia, while IL-6 remained elevated regardless of the disease state.

Brietzke et al. (2009) demonstrated that BD patients had increased IL-2, IL-4, and IL-6 during mania compared with healthy subjects. Meanwhile, patients with depressive episodes showed only increased IL-6 levels, and those in remission—only IL-4. In addition, the authors saw no difference in TNF-α levels between examined groups. Pietruczuk et al. (2019) showed that patients in remission had decreased serum TNF-α and increased IL-6 and IL-10 compared to healthy people. Also, the level of IL-17A was significantly lower in patients in depression or remission. Karthikeyan et al. (2022) showed no differences between BD patients and healthy people in serum IL-6 or TNF-α.

Increased levels of pro-inflammatory cytokine IL-1β and kynurenic acid have also been demonstrated in the cerebrospinal fluid of BD patients (Wang and Miller, 2018). In addition, in postmortem examinations, Rao et al. (2010) found that the expression of myeloid differentiation factor-88 (MyD88), nuclear factor-kappa B (NF-κB), proteins that are involved in the activation of the IL-1 pathway, were increased in the prefrontal cortex of BD patients.

In obese euthymic BD patients, higher sTNFR1 levels were accompanied by increased adipokines, adiponectin, and leptin (Barbosa et al., 2012). Among other pro-inflammatory factors, chemokine ligand 2 (CCL2) and pentraxin-related protein (PTX3) are worth mentioning. Drexhage et al. (2011) observed that serum levels of CCL2 and PTX3 were higher in euthymic BD patients than in healthy people. However, at the same time, the authors did not observe changes in the level of IL-1β, IL-6, or TNF-α.

Interesting results on the transcription of pro-inflammatory genes involved in the inflammation process, cell trafficking, or survival were shown by Padmos et al. (2008). For example, an increase in mitogen-activated protein kinases 6 (*MAPK6*) and *CCL2* was shown during the manic episode, while the depressive episode was accompanied by an increase in the transcription of *IL6*, *PTX3*, epithelial membrane protein 1 (*EMP1*), and BCL2 related protein A1 (*BCL2A1*) genes. Moreover, 55% of BD patients included in the study had increased transcription of the phosphodiesterase 4B (*PDE4B*) gene, which codes for a pro-inflammatory, cAMP-degrading enzyme expressed in immune cells; this enzyme has been proven to play an essential role in the pathophysiology of many psychiatric and neurological diseases (Fatemi et al., 2008; Padmos et al., 2008).

### 5.2. Changes in immune cells

Immunological changes in BD patients are seen in the number and ratio of selected immune cells. For example, a recent meta-analysis showed that BD patients had a higher neutrocyte/lymphocyte ratio than healthy subjects. In addition, the platelets/lymphocyte ratio was increased during manic episodes compared with healthy control and euthymic patients (Mazza et al., 2018). More recent studies have confirmed these findings by describing a higher monocyte/lymphocyte ratio as a predictor of manic episodes (Inanli et al., 2019; Kirlioglu et al., 2019).

Not many publications describe changes in the lymphocyte profile in BD patients. However, some alterations have been demonstrated, especially in a subpopulation of T cells (CD3^+^ cells). T cells are divided into helper T cells (CD4^+^ cells) and cytotoxic T cells (CD8^+^ cells). Naïve helper T cells can differentiate into many subpopulations, including Th1 cells responsible for cellular immune response, Th2 cells that regulate humoral responses, Th17 involved in pro-inflammatory reactions, or regulatory T cells (Tregs).

Drexhage et al. (2011) demonstrated no significant differences in the percentages of Th1, Th2, and Th17 cells between BD patients and healthy people. However, younger euthymic BD patients (less than 40 years old) had a higher percentage of regulatory (CD4^+^CD25^+^FoxP3^+^) T cells (Tregs) than healthy people of the same age. Also, BD patients with autoimmune thyroid disease (AITD) had a significantly reduced percentage of Tregs compared to BD patients without AITD. Increased levels of Tregs and Th17 cells in euthymic BD patients compared to healthy people were found in another study (Becking et al., 2018). Female euthymic patients with BD-I treated with mood stabilizers presented a reduced number of Tregs and a higher number of senescence-associated cytotoxic (CD8^+^CD28^−^) T cells compared to healthy controls (do Prado et al., 2013). Meanwhile, Barbosa et al. (2014) demonstrated a reduced percentage of cytotoxic T cells, an increased percentage of activated helper (CD4^+^CD25^+^) T cells, and a lower rate of CD4^+^CD25^+^FoxP3^+^IL-10^+^ Tregs in euthymic BD patients. Additionally, the authors saw increased percentages of monocytes (CD14^+^ cells). Also, reduced percentages of helper T cells expressing chemokine receptors in BD patients were demonstrated (Barbosa et al., 2019).

Changes in lymphocyte proportions also appear to depend on the phase of the disease. For example, Pietruczuk et al. (2019) showed that patients with BD-II had altered lymphocyte subpopulations depending on the disease phase. BD patients in depression and hypomania had a lower percentage of T cells compared to healthy control and patients in remission. On the other hand, patients in depression and remission had a higher percentage of B cells (CD19^+^ cells) than healthy people or patients in hypomania. Also, patients with depression and hypomania had a reduced percentage of CD4^+^ and CD8^+^ cells compared to healthy people. Euthymic patients also had a lower percentage of CD8^+^ cells than healthy control. Additionally, the authors saw that BD patients in hypomania had a lower percentage of activated helper T cells (CD4^+^CD25^+^) and activated cytotoxic T cells (CD8^+^CD25^+^) compared to patients in remission or depression. In contrast, patients in remission had a higher rate of CD8^+^CD25^+^ T cells compared to healthy people. Magioncalda et al. (2018) demonstrated that BD patients with mania presented a higher percentage of CD4^+^ cells, especially CD4^+^CD28^+^ T cells, and lower CD8^+^ cells, mainly CD8^+^CD28^−^ T cells, compared with healthy people.

Furlan et al. (2019) showed that total natural killer (NK) cell counts do not differ between BD-I patients and healthy people. However, patients had significantly higher circulating counts of NK cells producing INF-γ and IL-17.

## 6. Immunomodulation as a mechanism of lithium therapeutic action in bipolar disorder

Although various studies demonstrate the immunomodulatory effect of lithium, clinically relevant interpretation of the results might be complicated or even impossible, mainly due to the lack of a valid animal model for BD and the differing effect of Li on different cell populations. Only studies performed in BD patients and *in vitro* studies using human cells are included in this review.

### 6.1. Lithium influence on cytokines

Changes in IL-1β and IL-6 levels in BD patients treated with lithium were seen *ex vivo* and *in vitro*. For example, Guloksuz et al. (2010) showed that compared to healthy people and medication-free euthymic BD patients, euthymic patients on Li monotherapy had elevated serum TNF-α and IL-4 levels after 8 weeks of treatment. In another study, BD patients in immediate remission after mania had higher concentrations of IL-10 than healthy subjects and patients on sustained (minimum 6 months) remission on lithium therapy (Remlinger-Molenda et al., 2012). They also had higher IL-6 levels compared with patients in sustained remission. In addition, BD patients in remission after the depression had higher interferon-gamma (IFN-γ) concentrations than healthy people and patients in sustained remission. They also had higher TNF-α levels compared with patients in sustained remission. In general, the levels of cytokines in patients with sustained remission were similar to those observed in healthy people.

In BD patients treated with Li for a minimum of 6 months, monocytes stimulated with lipopolysaccharide (LPS) produced high IL-1β and low IL-6 levels compared to cells from non-treated patients (Knijff et al., 2007). However, *in vitro* exposure of monocytes to high (5 mM) concentrations of lithium chloride resulted in decreased production of IL-1β without any particular effect on IL-6 production. In healthy people, Li reduced the production of IL-6 and TNF-α and increased the secretion of IL-10 by cultured and activated human monocyte-derived dendritic cells (Leu et al., 2017). These observations would explain the differences in serum cytokine levels between patients in immediate and sustained remission demonstrated by Remlinger-Molenda et al. (2012).

Padmos et al. (2008) found that monocytes from lithium-treated BD patients had significantly lower gene expression of *TNF* and *PDE4B* than healthy controls and untreated BD patients. At the same time, lithium treatment caused a significant increase in the expression of *EMP1*, which induces apoptosis.

Another study measuring gene transcription in lymphocytes of BD patients treated with lithium showed changes in the expression of 56 genes (Anand et al., 2016). Among them, in comparison to those patients who did not receive Li, upregulation of several immunomodulation-related genes was shown, for example, the genes for IL-5 receptor (*IL5RA*), the interferon alpha-inducible protein (*IFI6*), and a regulatory factor affecting the expression of HLA class II (*RFX2*). In addition, genes for several protein kinases and phosphatases responsible for signal transduction, such as calcium/calmodulin-dependent protein kinase I (*CAMK1*), phosphoinositide-3-kinase (*PIK3R6*), and MOK protein kinase (*MOK*), were upregulated as well.

### 6.2. Lithium influence on immune cells

The latest study by Wu et al. (2019) revealed that patients with BD-I treated with lithium were characterized by higher percentages of total T cells, CD4^+^ T cells, activated B cells, and monocytes than healthy controls. *In vitro*, 5 and 10 mM of Li in the cell culture increased the percentage of monocytes and dendritic cells. Pietruczuk et al. (2018) showed that lymphocytes of BD patients treated with lithium for at least 6 months had reduced proliferation capacity reflected as a decreased percentage of cells proliferating in response to *in vitro* stimulation and accompanied by increased susceptibility to apoptosis. However, *in vitro*, Li protected patients’ lymphocytes from apoptosis proportionally to the dose (1–2.5 mM) without significantly influencing their proliferation capacity.

Several studies were performed on lymphoblastoid cell lines (LCL) derived from BD patients in which the apoptosis-regulating effect of lithium has been demonstrated. Overall, the presence of lithium in cell culture altered the expression of 236 genes, most of which were responsible for regulating apoptosis (Fries et al., 2017). Another study performed on LCL of BD patients showed that lithium, in both lithium-responders and non-responders-derived cells, downregulated the Let-7 family genes coding microRNAs (miRNAs) playing a role in post-transcriptional changes in immune system cells, influencing both innate and adaptive immune response (Hunsberger et al., 2015).

Interestingly, two studies have underlined the significant role of insulin-like growth factor 1 (IGF-1) in lithium response in BD patients. IGF-1 primary role in CNS is the promotion of neuronal survival by inhibition of apoptosis. IGF-1 also affects cell signaling and neurotransmission (Bhalla et al., 2022). Genetic studies have suggested that the *IGF1* gene might play a significant role in the pathophysiology of bipolar disorder (Pereira et al., 2011). Squassina et al. (2013) found that the *IGF1* gene was significantly overexpressed in BD patients taking lithium but only in those responding to therapy. Another study by Milanesi et al. (2015) showed that exogenous IGF-1 added to LCL culture *in vitro* increased lithium sensitivity in cells from non-responding BD patients – the growth of cells was inhibited in the presence of IGF-1 and 10 mM of Li.

**Figure 2:**
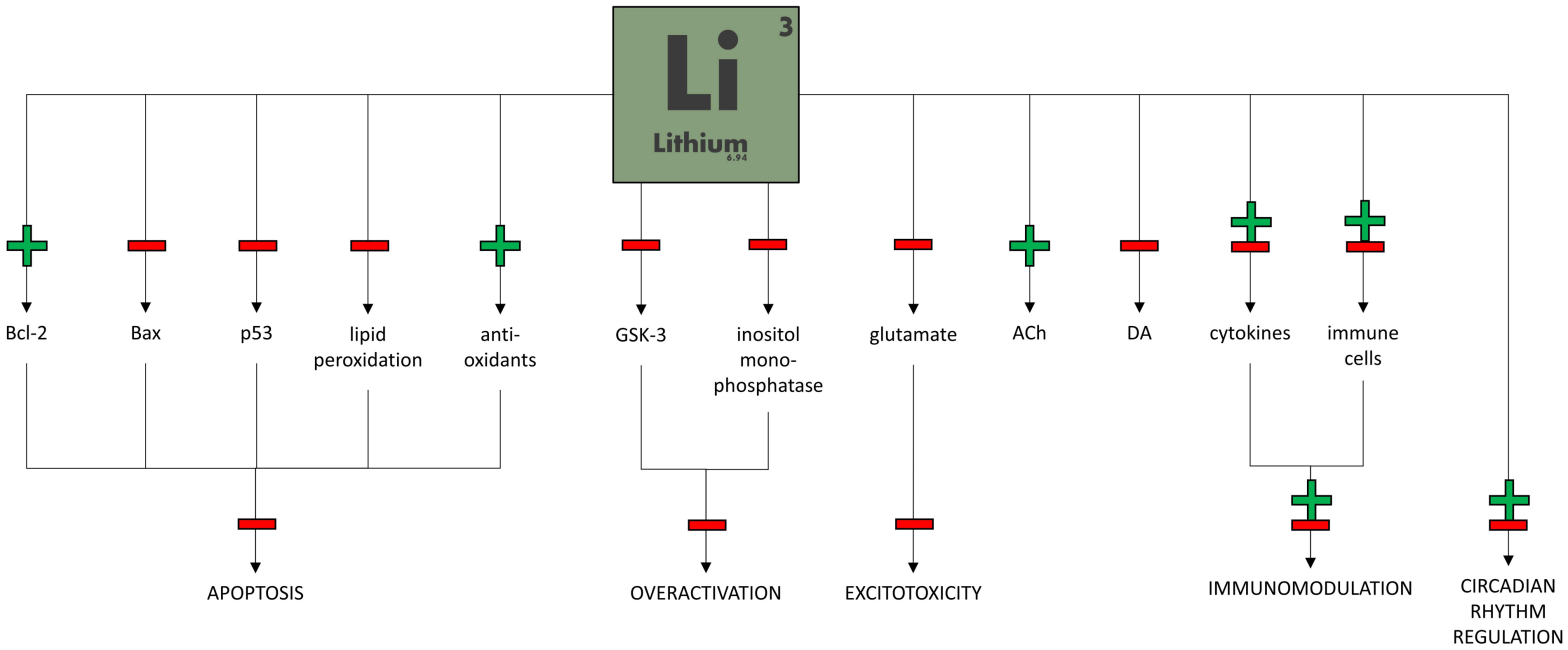
Potential mechanisms of lithium therapeutic action in bipolar disorder.

Figure 2 shows the potential mechanisms of lithium therapeutic action in bipolar disorder.

## 7. Limitations of the studies

The authors of the reviewed studies pointed out several limitations of their research. Most agree that study groups were not big enough to make unified, robust conclusions about lithium immunomodulatory actions in bipolar patients. Anand et al. (2016) pointed out that a small number of subjects in the mania and depression subgroups made it difficult to identify differences between the effects of Li in the two states. Remlinger-Molenda et al. (2012) underlined that patients in their study were not controlled for medication use after immediate remission. Also, there was a significant age difference between groups of post-depression euthymic and post-manic euthymic patients. The research group did not exclude other factors that may be associated with increased systemic inflammation, such as cigarette smoking, alcohol use, weight gain, physical activity, and quality of sleep as well as psychological stress, both acute and chronic.

A significant limitation of many studies cited here is using peripheral lymphocyte gene expression and cytokine levels in the blood, which may not fully reflect the ones in the central nervous system. Wu et al. (2019) indicated the possible effects of the concomitant administration of psychotropic drugs on immunological endophenotypes. Many studies used healthy subjects as the control group due to difficulties in including drug-naive BD patients. Finally, it is essential to underline that *in vitro* findings might not fully correspond to the real-life effects of lithium.

## 8. Conclusion

The pathophysiology of bipolar disorder is still not fully understood. While environmental and genetic factors play an essential role in the pathogenesis of BD, immunological disturbances were also described. Lithium, one of the oldest and the most efficient treatment for BD, has a variety of mechanisms of action. Its modulatory effect on several genes regulating neurotransmission, neuromodulation, neuroplasticity, circadian rhythm, and inflammatory response has been shown. However, its immunomodulatory properties are still poorly recognized.

In BD patients, Li influences the production of several pro- and anti-inflammatory cytokines, and this effect seems to be specific for the phase of the disease as well as the length of the treatment. Several immunomodulatory, apoptotic, and kinase-coding genes seem to be unregulated and positively correlated with good lithium response. Increased prevalence of CD4^+^ T cells and activation of B cells and monocytes were also observed. Lower susceptibility to apoptosis of lymphocytes cultivated with the presence of Li was also described. Moreover, higher expression of IGF-1, an anti-apoptotic agent, might play an essential role in good lithium treatment outcomes. Unfortunately, the results of studies are often incoherent. It might be caused by the presence of many variables affecting the immune system of BD patients, including the phase and duration of the disease, the length of the episode, misdiagnosis, age, gender, and the effects of other substances that can also modulate the immune system.

According to the assumptions of psychoneuroimmunology, impaired regulation of immune system activity and chronic inflammation may play an essential role in the pathogenesis of bipolar disorder. Moreover, numerous studies indicate that the treatment of depressive disorders, including bipolar disorder, can be effective by modifying the immune response, for example, through the use of non-steroidal anti-inflammatory drugs (NSAIDs). Therefore, further research is needed to understand the connection between immune system modulation and the therapeutic action of lithium in BD. Future studies on the immunomodulatory mechanism of lithium’s actions and other mood stabilizers and psychiatric medications might contribute to a new approach to treating psychiatric conditions based on immune system regulation.

## Author contributions

ŁS, MB, and GP contributed to the conception of the review and wrote the first draft of the manuscript. ŁS organized the database. KL and WC reviewed, corrected, and edited the manuscript. ŁS and KL prepared the figure. All authors contributed to the article and approved the submitted version.

## Funding

This work was supported by the statutory funds of the Medical University of Gdańsk (no. 02-0039/07/221, granted to WC).

## Conflict of interest

GP was a consultant to Lundbeck, Angelini, and FB-Health and received scholarship/research support from Lundbeck and Angelini. In addition, he is a member of the speaker/advisory board of Sanofi-Aventis, Lundbeck, FB-Health, and Angelini. WC received grants from Acadia, Alkermes, Allergan, Angelini, Auspex Pharmaceuticals, BMS, Celon, Cephalon, Cortexyme, Ferrier, Forest Laboratories, Gedeon Richter, GWPharmaceuticals, HMNC Brain Health, IntraCellular Therapies, Janssen, KCR, Lilly, Lundbeck, Minerva, MSD, NIH, Novartis, Orion, Otsuka, Sanofi, Servier, honoraria from Adamed, Angelini, AstraZeneca, BMS, Celon, GSK, Janssen, KRKA, Lekam, Lundbeck, Minerva, NeuroCog, Novartis, Orion, Pfizer, Polfa Tarchomin, Sanofi, Servier, Zentiva. He was on the advisory boards of Angelini, Celon (terminated), Douglas Pharmaceuticals, Janssen, MSD, Novartis, and Sanofi.

The remaining authors declare that the research was conducted in the absence of any commercial or financial relationships that could be construed as a potential conflict of interest.

## Publisher’s note

All claims expressed in this article are solely those of the authors and do not necessarily represent those of their affiliated organizations, or those of the publisher, the editors and the reviewers. Any product that may be evaluated in this article, or claim that may be made by its manufacturer, is not guaranteed or endorsed by the publisher.
